# Sequence characteristics, expression and subcellular localization of *PtCYP721A57* gene from cytochrome P450 family in *Polygala tenuifolia* willd

**DOI:** 10.7717/peerj.18089

**Published:** 2024-10-18

**Authors:** Yao Luo, Benxiang Hu, Haiyue Ji, Yiyao Jing, Gang Zhang, Yonggang Yan, Bingyue Yang, Liang Peng

**Affiliations:** College of Pharmacy, Shaanxi University of Chinese Medicine/Shaanxi Engineering Research Center for Application and Development of Chinese Medicine in Qinling Mountains/Key Laboratory of “Qin Medicine” Research and Development, Xianyang, China

**Keywords:** *Polygala tenuifolia*, Cytochrome P450, Gene cloning, Subcellular localization, Gene expression

## Abstract

The Cytochrome P450 (CYP450) family is the largest enzyme protein family in plants, distributed across various organs and involved in significant catalytic activities in primary and secondary metabolic processes. In this study, we cloned the *PtCYP721A57* gene, characterized its open reading frame (ORF), and conducted comprehensive analyses including physicochemical properties, evolutionary relationships, subcellular localization, prokaryotic expression, and correlation between the relative expression of different parts and the content of tenuifolin, hormones, and abiotic stress response associated with the encoded protein. The ORF of *PtCYP721A57* was 1,521 bp, with a secondary structure predominantly composed of α-helices and random coils. Subcellular localization experiments confirmed the presence of *PtCYP721A57* in the endoplasmic reticulum. For prokaryotic expression, we constructed the recombinant plasmid pET28a-*PtCYP721A57* using pET28a as the vector, which was then transformed into BL21(DE3). Induction with Isopropyl β-D-1-thiogalactopyranoside (IPTG) at temperatures of 16 and 25 °C and varying concentrations (0.1, 0.2, 0.5, 1, 2 mM) resulted in the formation of inclusion bodies, with higher expression observed at 25 °C. Our qPCR analyses revealed that *PtCYP721A57* exhibited the highest expression in the cortex of *Polygala tenuifolia*, followed by roots and xylem, correlating with the observed tenuifolin content distribution. Induction with abscisic acid (ABA) and chitosan (CHT) initially decreased *PtCYP721A57* expression followed by a subsequent increase, peaking at 48 h. Similarly, drought stress induced a gradual increase in *PtCYP721A57* expression, also peaking at 48 h. NaCl treatment for 6 h significantly upregulated *PtCYP721A57* expression. In conclusion, our study provides foundational insights into the *PtCYP721A57* gene in *Polygala tenuifolia*, laying the groundwork for further exploration of its role in the biosynthesis pathway of triterpenoid saponins.

## Introduction

Polygala Radix, a traditional Chinese medicine derived from *Polygala tenuifolia* Willd., is characterized by its “bitter, hot, and warm properties”, and “belongs to the heart, kidney, and lung meridians”. Originally documented in the “*Shennong Herbal Classics”*, it is renowned in the Chinese Pharmacopeia for its “tranquilizing, enlightening, phlegm-dispelling, and swelling-reducing effects.” Clinically, it is used for palpitations, insomnia, amnesia, epilepsy, cough with phlegm, carbuncles, sore toxins, and breast swelling and pain (Sun et al., 2023). This medicinal herb is among the 85 traditional bulk medicinal materials for export and is one of 42 tertiary wild varieties under key protection in China (Xue et al., 2015). It ranks among the top three single-flavor Chinese herbs in clinical nootropic prescriptions and is considered essential for health maintenance. Studies have identified over 140 compounds from *P. tenuifolia*, primarily including saponins, ketones, oligosaccharide esters, alkaloids, and other bioactive components. These compounds and extracts exhibit a broad spectrum of pharmacological activities, such as anti-aging, neuroprotective, antidepressant, sedative-hypnotic, anti-inflammatory, antiviral, anticancer, antioxidant, and antiarrhythmic effects (Yao et al., 2022). *P. tenuifolia* is widely distributed in the Northwest, North, and Northeast regions of China, with particularly abundant and high-quality sources found in Shaanxi and Shanxi Provinces. It is classified as a moderate xerophyte with strong drought resistance but susceptibility to waterlogging (Peng et al., 2018).

The primary active ingredient in *P. tenuifolia* is oleanane-type triterpenoid saponins, which exhibit pharmacological actions such as enhancing learning and memory, sedation, and antidepressant effects (Lacaille-Dubois, Delaude & Mitaine-Offer, 2020). In higher plants, the biosynthesis of oleanane triterpenoids occurs in three stages: precursor supply, skeleton synthesis, and terpenoid synthesis. During the third stage, β-amyrin synthase (*β*-AS) catalyzes the conversion of 2,3-oxidosqualene (OS) to β-amyrin. Under the influence of Cytochrome P450 (CYP450) enzymes, *β*-amyrin undergoes oxidation at C-28 to form oleanolic acid. Subsequently, oleanolic acid is further modified by CYP450s and UDP-glucuronosyltransferases to produce a diverse array of triterpenoids (Sandeep et al., 2019; Fanani et al., 2019).

CYP450 is the largest family of enzymes in plants (Liu et al., 2022), characterized by a cysteine-iron heme structure that enables the reduction of oxygen molecules and a wide array of catalytic activities. It plays a crucial role in the biosynthesis of terpenoids, alkaloids, and phytohormones (Yu et al., 2017). Structural modifications catalyzed by CYP450 are pivotal for the diversification and functionalization of triterpenoid saponins, making it a key enzyme in the biosynthetic pathway of these components (Mak & Denisov, 2018). P450 genes also contribute to plant growth and development, enhancing tolerance to both biotic and abiotic stressors (Laffaru & Sharma, 2021).

Yang et al. (2018) demonstrated that the CYP450 enzyme in *Betula platyphylla* exhibits tissue-specific and hormone-induced expression characteristics, potentially playing a crucial role in the growth, development, stress resistance, and metabolite synthesis of *Betula platyphylla*. The identification and functional verification of key *CYP450* genes involved in the biosynthetic pathway of triterpenoid saponins in *P. tenuifolia*, along with the application of plant metabolic engineering to enhance triterpenoid saponin content, have significant theoretical and practical implications. This study utilized bioinformatics analysis of *PtCYP721A57*, combining protein subcellular localization, prokaryotic expression, response to hormones and abiotic stress, relative expression across different plant parts, and accumulation of tenuifolin content to elucidate its role in the biosynthesis pathway of tenuifolin. It may provide insights into the crucial role of the *CYP450* gene in the defense responses of *P. tenuifolia*, offer evidence for understanding the biosynthesis pathway and regulatory mechanisms of tenuifolin components, and establish a foundation for enhancing the quality of medicinal materials through genetic engineering. This research laid a basis for further investigations into the functional aspects of the *CYP450* gene in *P. tenuifolia*.

## Materials and Methods

### Experimental materials

*P. tenuifolia* seeds and plants were collected from the Pharmaceutical Botanical Garden of Shaanxi University of Traditional Chinese Medicine (Xianyang, Shaanxi Province) (108°74′E, 34°31′N). Three strains of 3-year-old *P. tenuifolia* with consistent growth were selected, and the roots, stems, and leaves were pooled for full-length transcriptome sequencing analysis. Additionally, different parts (roots, xylem, cortex) were collected under uniform conditions for further study, in conjunction with previous experimental results from our research group and related studies. Thirty-day-old seedlings of *P. tenuifolia*, showing robust growth and uniform developmental status, were selected for the following treatments:

Seedlings were exposed to 200 μmol·L^−1^ chitosan (CHT), 200 μmol·L^−1^ abscisic acid (ABA), 100 mmol·L^−1^ NaCl, and 10% PEG6000 for durations of 0, 6, 12, 24 and 48 h. Untreated seedlings served as the blank control.

### Amplification of the *PtCYP721A57*

The total RNA from *P. tenuifolia* seedlings was extracted using the Trizol extraction kit from Bioengineering Co., Ltd. (Shanghai, China), following the manufacturer’s protocol. The extracted RNA was then reverse transcribed into cDNA using the PrimeScript^™^ II 1st strand cDNA Synthesis Kit with Reverse Transcriptase from Takara Co., Ltd. (Japan). Specific primers designed for the *PtCYP721A57* gene sequence (CYP721A57-1-F/R, as listed in Table 1) were used for PCR amplification with the cDNA as a template. The resulting PCR products were analyzed by 1.0% agarose gel electrophoresis. The target DNA fragment was purified and recovered using a DNA gel recovery kit, ligated with pMD^™^19-T vector and transformed into DH5α competent cells. Positive clones were selected and sent to Aoke Biotechnology Co., Ltd. (Yangling, China) for sequencing.

**Table 1:** Primer sequences for gene cloning and expression analyses of *PtCYP721A57*.

Primer	Sequence (5′→3′)	Usage	Length/bp
PtCYP721A57-1	F: ATGATGCAGTTCCTCTTAGCT	ORF amplification	1,521
	R: TCAATCACGAAGTTTCGTGA		
PtCYP721A57-2	F: CGGGATCCATGATGCAGTTCCTCTTAGCTG	ORF amplification of prokaryotic expression	1,521
	R: CCCAAGCTTTCAATCACGAAGTTTCGTGAAGATG		
q-PtCYP721A57	F: AAGGGATCAAAGGACCTGGC	q-PCR	152
	R: TGGTGCCCACTTGTGGTAAA		
GADPH	F: ACAGCAACGTGCTTCTCACC	q-PCR	128
	R: CCCTTCATCACCACCGACTA		

### Bioinformatics analysis of the *PtCYP721A57* protein

Bioinformatics analysis and prediction of *PtCYP721A57* were conducted using online bioinformatics software tools (Table 2).

**Table 2:** Bioinformatics software and websites for analyses of *PtCYP721A57*.

Biological information	Related software and website
Open reading frame	https://www.ncbi.nlm.nih.gov/
Prediction of physical and chemical properties	ProtParam (https://web.expasy.org/protparam/)
Phosphorylation site prediction	NetPhos 3.1 Serve (https://services.healthtech.dtu.dk/services/NetPhos-3.1/)
Protein structural domain prediction	ExPASy (https://prosite.expasy.org/prosite.html)
Prediction of secondary structure	SOPMA (https://npsa-prabi.ibcp.fr/cgi-bin/npsa_automat.pl?page=npsa%20_sopma.html)
Prediction of tertiary structure	SWISS-MODEL (https://swissmodel.expasy.org/interactive/)
Protein signal peptide prediction	SignalP-5.0 (https://services.healthtech.dtu.dk/service.php?SignalP-5.0)
Transmembrane information	TMHMM (https://services.healthtech.dtu.dk/service.php?TMHMM-2.0)
Phylogenetic tree construction and beautification	MEGA11 Chiplot (https://www.chiplot.online/tvbot.html)

### Prokaryotic expression analysis of *PtCYP721A57* protein

For prokaryotic expression of *PtCYP721A57*, the primer pair *CYP721A57*-2-F/R (Table 2) was used for polymerase chain reaction (PCR) amplification, introducing BamHI and HindIII restriction sites upstream and downstream of the gene. After purification and recovery of the target fragment using a DNA gel recovery kit, the fragment was ligated into the pMD19-T vector and transformed into *E. coli* DH5α competent cells. Positive clones were selected and sequenced by Aoke Biotechnology Co., Ltd., Beijing, China. The *PtCYP721A57* gene fragment and the pET28 vector were digested with BamHI and HindIII, respectively. The resulting gene and vector fragments were recovered and ligated overnight at 16 °C. The recombinant plasmid was then transformed into *E. coli* BL21 (DE3) cells. After confirmation by sequencing, monoclonal cells were selected and cultured at 37 °C with shaking at 200 rpm until reaching an OD600 of 0.6–0.8 (approximately 12 h). Subsequently, the recombinant protein and the corresponding empty vector bacterial solutions were divided into two groups and cultured at different temperatures (16 and 25 °C). Each temperature group included an empty vector control, a non-induced recombinant protein group, and groups induced with varying concentrations of Isopropyl β-D-1-thiogalactopyranoside (IPTG: 0.1, 0.2, 0.5, 1, 2 mM). Following induction, 1–2 mL samples were collected for subsequent sodium dodecyl sulfate-polyacrylamide gel electrophoresis (SDS-PAGE) analysis. The remaining bacterial solution was centrifuged at room temperature to collect cells (5,000 rpm, 6 min). The cells were then resuspended in 10 mL phosphate-buffered saline (PBS) and subjected to two additional rounds of centrifugation and resuspension in PBS. Cell disruption was achieved on ice for 10 min using a high-pressure disruptor. The lysate was then centrifuged at 4 °C (12,000 rpm, 20 min), and 1 mL of the supernatant was collected for SDS-PAGE analysis.

### Quantitative real-time polymerase chain reaction (qRT‑PCR) analysis

The expression levels of the *PtCYP721A57* gene in different parts of *P. tenuifolia* and its response to NaCl, PEG6000 stress, and exogenous CHT and ABA treatments were analyzed using qRT-PCR. The primer pair q-*PtCYP721A57*-F/R (Table 1) was used for PCR amplification. Relative expression of *PtCYP721A57* in each plant part and under various treatment time points was determined based on the Ct value at the plateau stage of quantitative amplification curves. Glyceraldehyde-3-phosphate dehydrogenase (GAPDH) was a normalization reference gene (Gao et al., 2012). The StepOnePlus^™^ Real-Time PCR instrument (Applied Biosystems, Waltham, MA, USA) was employed for these analyses. The relative expression level of the *PtCYP721A57* gene was calculated using the 2^−ΔΔCt^ method (Li et al., 2019).

### Statistical analysis

Image processing and t-test analysis were conducted using Prism 9.0 (GraphPad Software, La Jolla, CA, USA) and SPSS 27.0 (IBM Corp., Armonk, NY, USA) software packages. A significance level of *P* < 0.05 was used to determine statistical significance.

## Results

### Cloning of the *PtCYP721A57* gene

**Figure 1:**
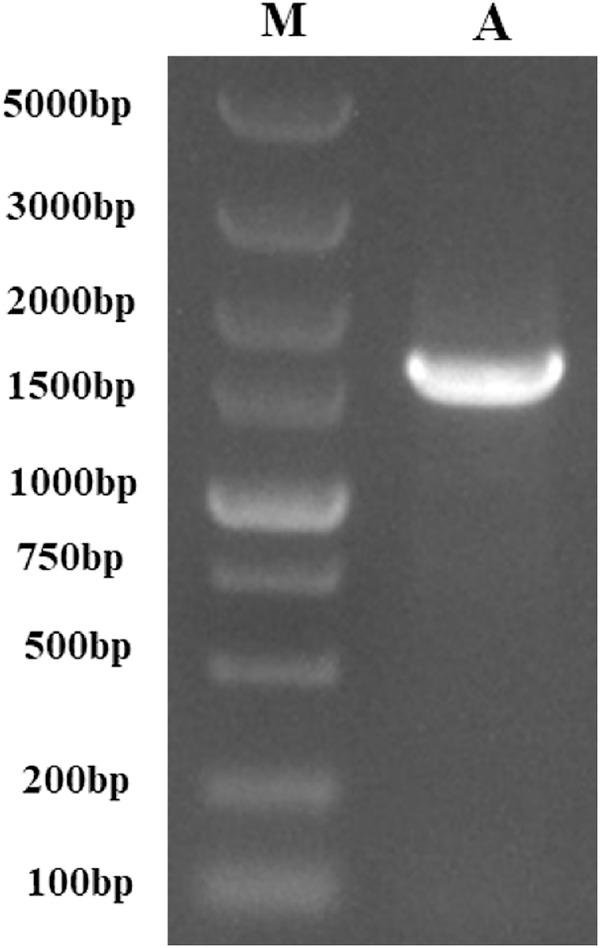
The PCR-amplified product of *PtCYP721A57*. M, Gene Ruler DNA Ladder Mix; A, PCR amplified product of *PtCYP721A57*.

**Figure 2:**
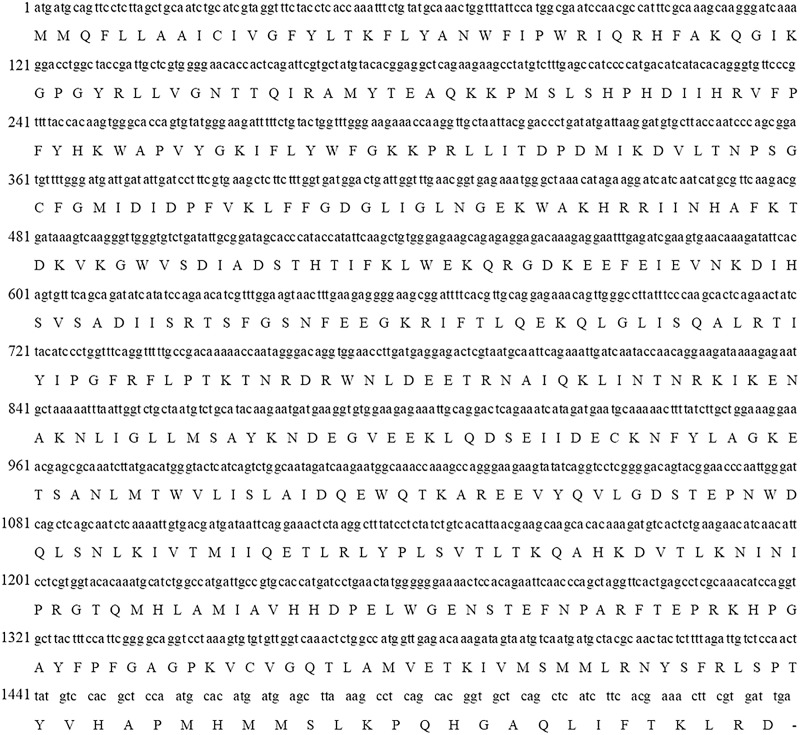
Complete cDNA and deduced amino acid sequences of *PtCYP721A57* gene.

**Figure 3:**
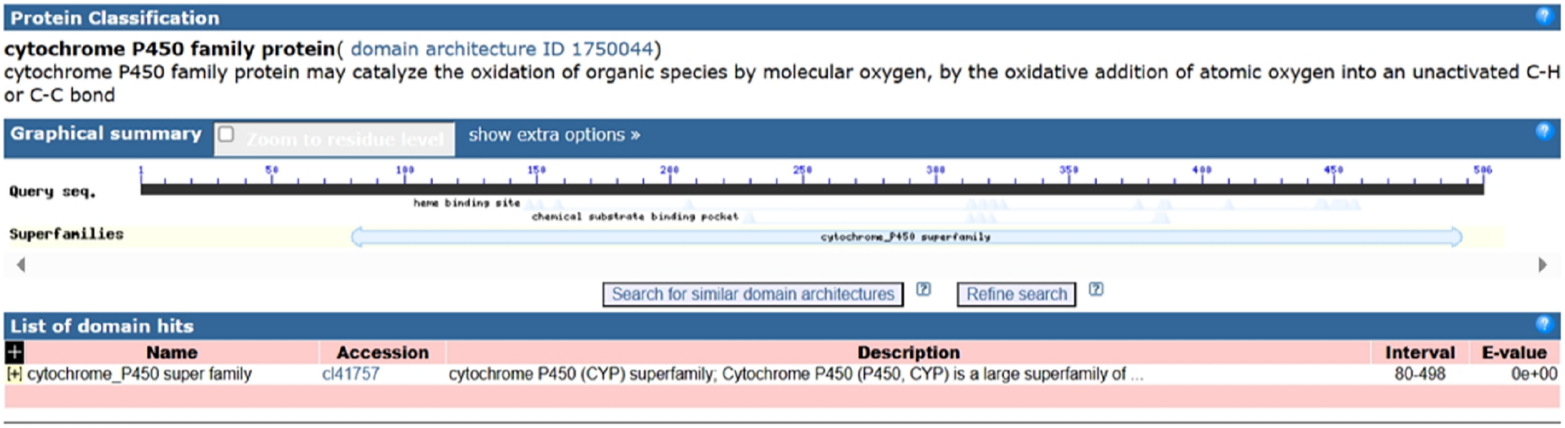
Domain analysis of PtCYP721A57 protein.

Following the sequencing and annotation of the full-length transcriptome of *P. tenuifolia*, a *PtCYP450* gene was identified. Subsequent PCR amplification, sequencing analysis, and NCBI alignment confirmed the presence of the *P. tenuifolia* gene *CYP721A57*. This gene possessed an open reading frame (ORF) of 1,521 base pairs, encoding a 506-amino acid protein and contained a conserved CYP450 domain, thereby confirming its classification within the CYP450 family (Figs. 1–3).

### Structural analysis of *PtCYP721A57* protein

The protein encoded by PtCYP721A57 underwent a comprehensive analysis, which included determination of its relative molecular mass, isoelectric point (pI), hydrophilicity, prediction of transmembrane domains, and identification of phosphorylation sites.

The protein encoded by PtCYP721A57 had a molecular formula of C_2665_H_4149_N_713_O_728_S_22_, totaling 8,277 atoms, and a relative molecular mass of 58.54 kDa. It consisted of a polypeptide chain comprising 506 amino acids. The theoretical isoelectric point (pI) of the protein was calculated to be 9.30, indicating it was alkaline. Additionally, the protein exhibited a lipid index of 64.97 and an instability index (II) of 53.06. Analysis of amino acid hydrophobicity (Fig. S1) revealed an average hydrophilicity coefficient of −0.312 and an instability coefficient of 32.40, suggesting stability as a hydrophobic protein. Understanding the phosphorylation sites and levels of the target protein can provide an important reference for characterizing its properties (Cong, 2020). Prediction of phosphorylation sites in the PtCYP721A57 protein indicated multiple sites, with 23 serine, 31 threonine, and 15 tyrosine (Fig. S2).

**Figure 4:**
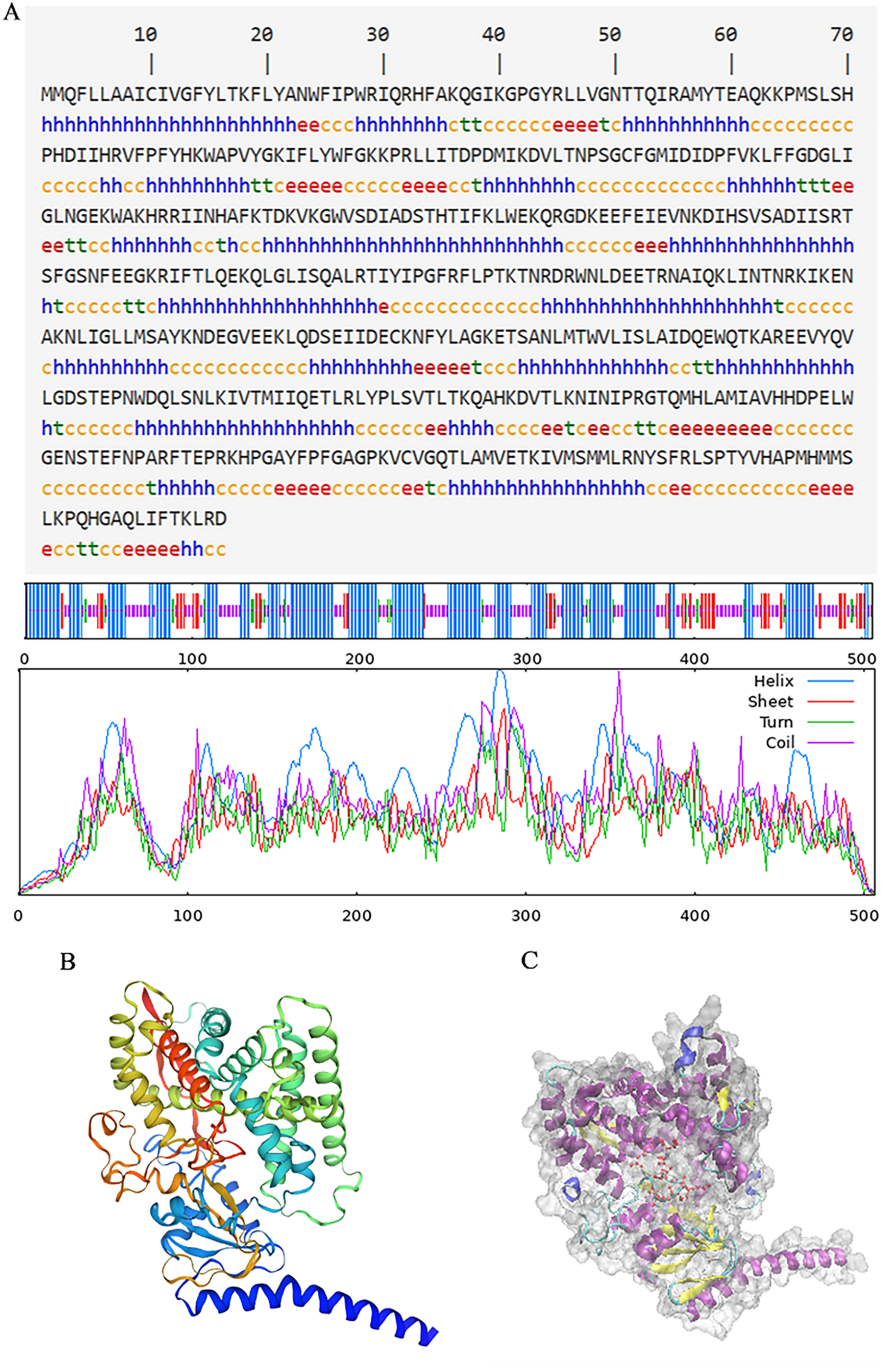
Structure prediction of PtCYP721A57. (A) Secondary structure model of PtCYP721A57; (B) prediction of the tertiary structure of PtCYP721A57 protein; (C) tertiary model of PtCYP721A57 protein C: α-helix (purple); extension chain (yellow); β-turn (cyan); random coil (blue); heme domain (red).

The secondary structure of proteins, characterized by the arrangement of the polypeptide backbone atoms, encompassed α-helices, β-sheets, β-turns, and random coils. Predicting this structure provides insights into the relationship between protein structure and its function (Li et al., 2022). For the PtCYP721A57 protein, the predicted secondary structure includes 49.01% α-helix, 12.25% β-sheet, 5.34% extended chain, and 33.40% irregular coil structures, with α-helices being the predominant element (Fig. 4A). Additionally, the three-dimensional structure of PtCYP721A57 was analyzed using SWISS-MODEL online software, employing cytochrome P4504B1 (SMTL ID: 6c94.1) as a template. The analysis indicated 100% coverage of the protein structure (Figs. 4B and 4C).

### Prokaryotic expression analysis

The *PtCYP721A57* gene and the pET28a vector underwent double digestion, followed by ligation using T4 ligase. After PCR verification, the target gene was sequenced, and analysis confirmed that the sequence matched the original, indicating the successful construction of the prokaryotic expression vector pET28a-PtCYP721A57.

**Figure 5:**
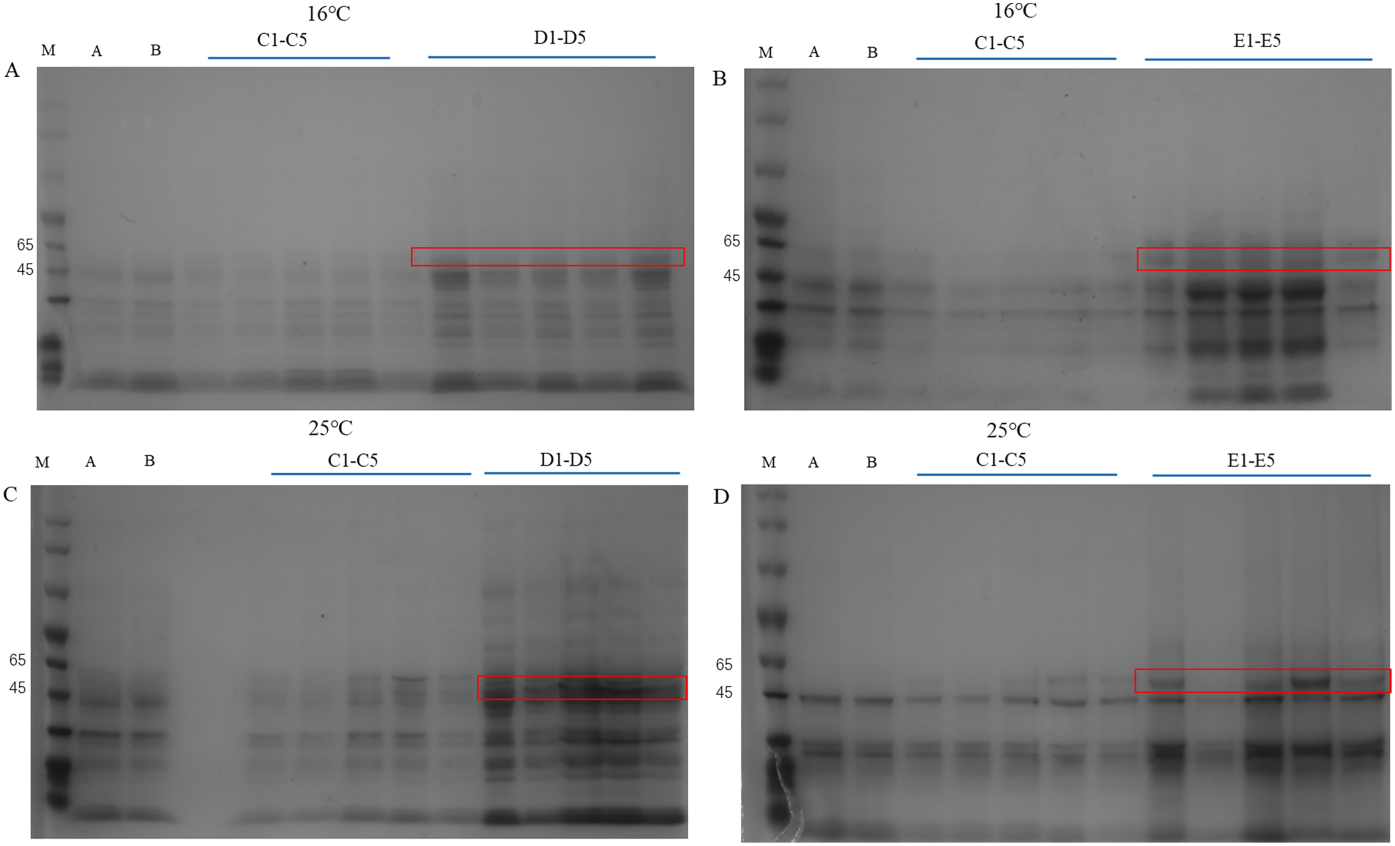
Detection of PtCYP721A57 recombinant protein expressed by different temperatures and IPTG concentration. (A–D) M, Marker; A, pET28a-BL21(DE3); B, pET28a-PtCYP721A57-BL21(DE3); C1–C5, different concentrations of IPTG induced recombinant vector bacterial solution; D1–D5, Supernatant; E1–E5, urea dissolution precipitation.

The pET28a-PtCYP721A57 recombinant vector was introduced into BL21(DE3) cells, with strains transformed using the empty pET28a vector serving as controls. Different concentrations of IPTG were used to induce protein expression. SDS-PAGE analysis following cell disruption (Fig. 5) revealed that compared to both the control groups (blank and non-induced), IPTG significantly stimulated the expression of the recombinant protein. Differences in IPTG concentrations did not significantly affect protein expression, confirming the successful expression of the recombinant plasmid. However, no protein expression was observed in the supernatant, suggesting the absence of soluble protein. The addition of 8M urea facilitated overnight dissolution of the precipitate at 4 °C, leading to detectable expression, and confirming the formation of inclusion bodies. Additionally, it was found that expression in inclusion bodies was higher at 25 °C compared to 16 °C.

### Subcellular localization analysis

**Figure 6:**
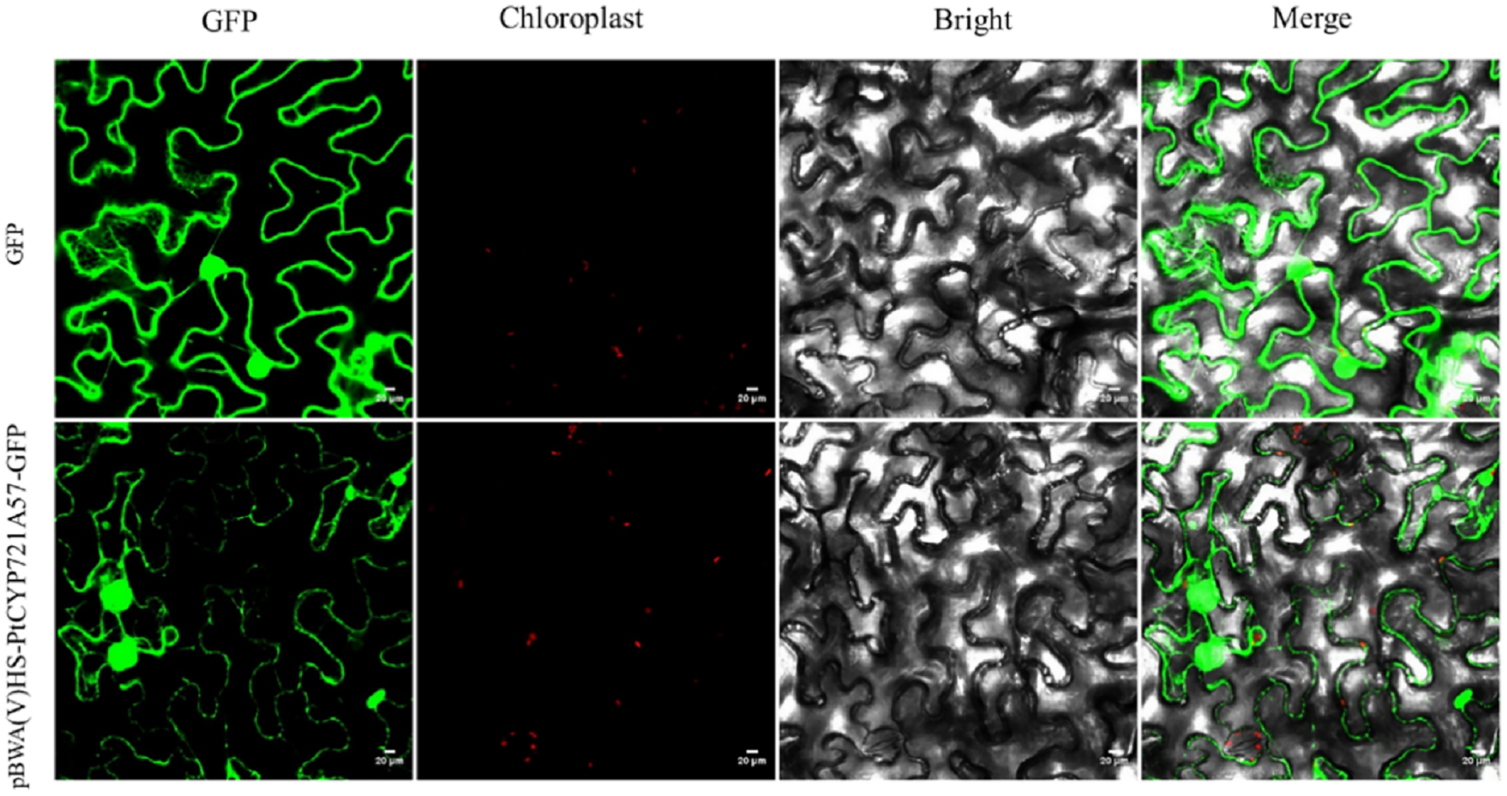
Subcellular localization of PtCYP721A57 protein in lower epidermal cells of tobacco leaves.

To explore the cellular role of the PtCYP721A57 protein, the recombinant plasmid pBWA(V)HS-*PtCYP721A57*-GLosgfp was constructed. The localization of the protein within cells was assessed using tobacco transient transformation. Sequencing confirmed the successful ligation of the target gene with the vector, validating the construction of the recombinant subcellular localization vector. Observation of tobacco leaf epidermal cells under a laser confocal microscope revealed green fluorescence signals distributed in the endoplasmic reticulum from the pBWA(V)HS-PtCYP721A57-GLosgfp recombinant vector (Fig. 6).

### Phylogenetic tree analysis of protein sequences

**Figure 7:**
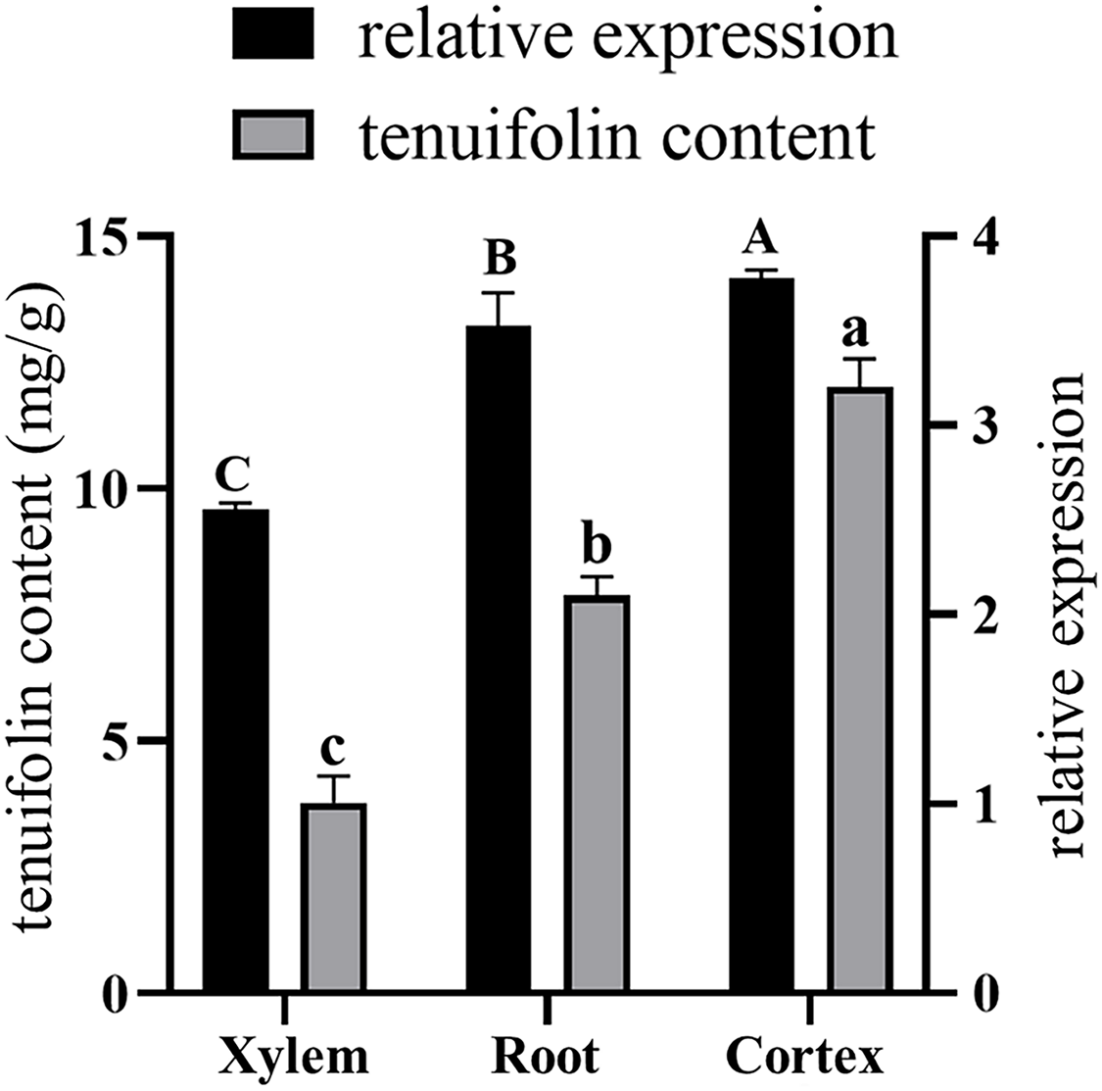
Phylogenetic analysis of *PtCYP721A57*.

In this study, the amino acid sequences of CYP450 family members from various plants, including *Arabidopsis thaliana* (a model plant), and those sharing homology with *PtCYP721A57* from Teleostei were retrieved from the NCBI database. Using MEGA 11.0 software, a phylogenetic tree was constructed to analyze the relationship among *PtCYP721A57* and other CYP450 family members (Fig. 7). The phylogenetic analysis revealed that proteins encoded by CYP72 subfamily genes formed a distinct branch. This branch showed high homology with sequences from *P. tenuifolia*, *Juglans regia*, and Quercus. This clustering suggests potential similar biological functions among the proteins within this branch.

### Gene expression analysis

**Figure 8:**
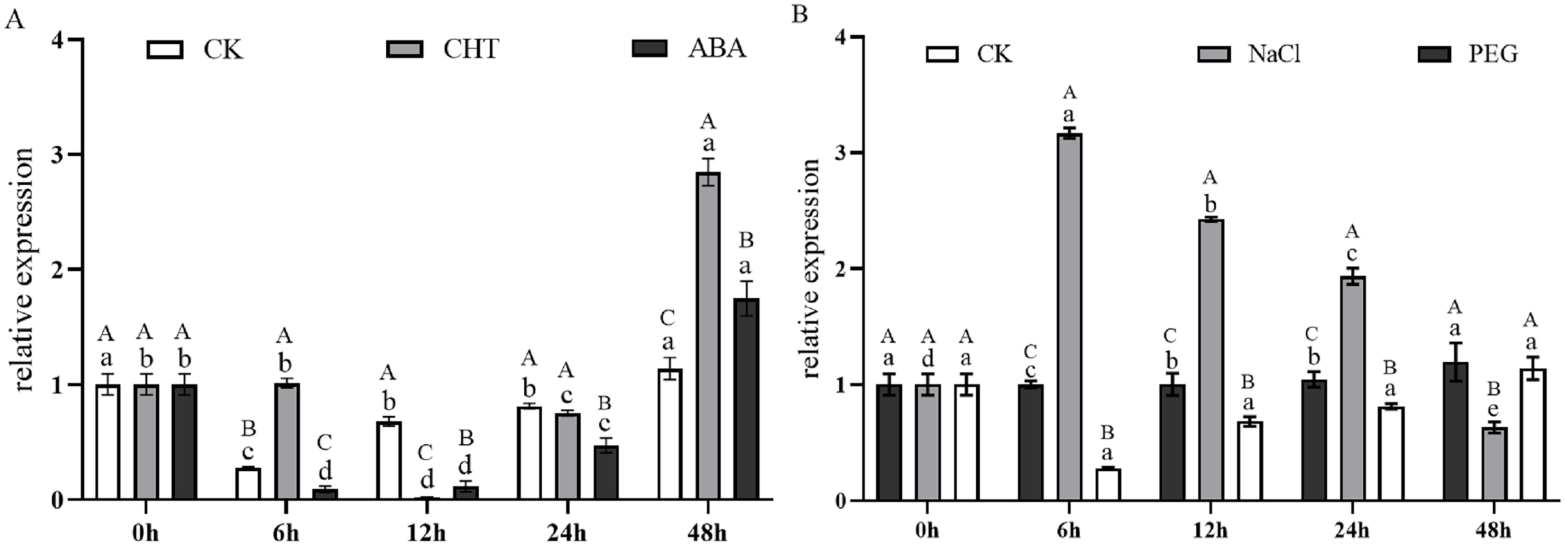
The correlation analysis between the relative expression of the *PtCYP721A57* gene in different tissues and the content of tenuifolin was analyzed. Different capital letters above the column represent the content of tenuifolin in other tissues, and different lowercase letters represent the relative expression levels in different tissues with significant statistical differences (*P* < 0.05).

Total RNA was extracted from the roots, xylem, and cortex of *P. tenuifolia*, and qRT-PCR was used to evaluate the expression of the *PtCYP721A57* gene in each tissue. The GADPH gene (housekeeping gene) served as a reference for normalizing *PtCYP721A57* expression levels (Fig. 8). Using the equation y = 341,572x − 156,068, the tenuifolin content was calculated for each sample. The cortex exhibited the highest tenuifolin content, followed by the root, while the xylem showed the lowest content (9.594 ± 0.122 mg/g). When using the xylem sample as a reference, the relative expression of the *PtCYP721A57* gene correlated with the change in tenuifolin content. Correlation analysis revealed a significant positive correlation between the relative expression of *PtCYP721A57* and tenuifolin content, with a correlation coefficient of 0.900 (*P* **<** 0.01).

**Figure 9:**
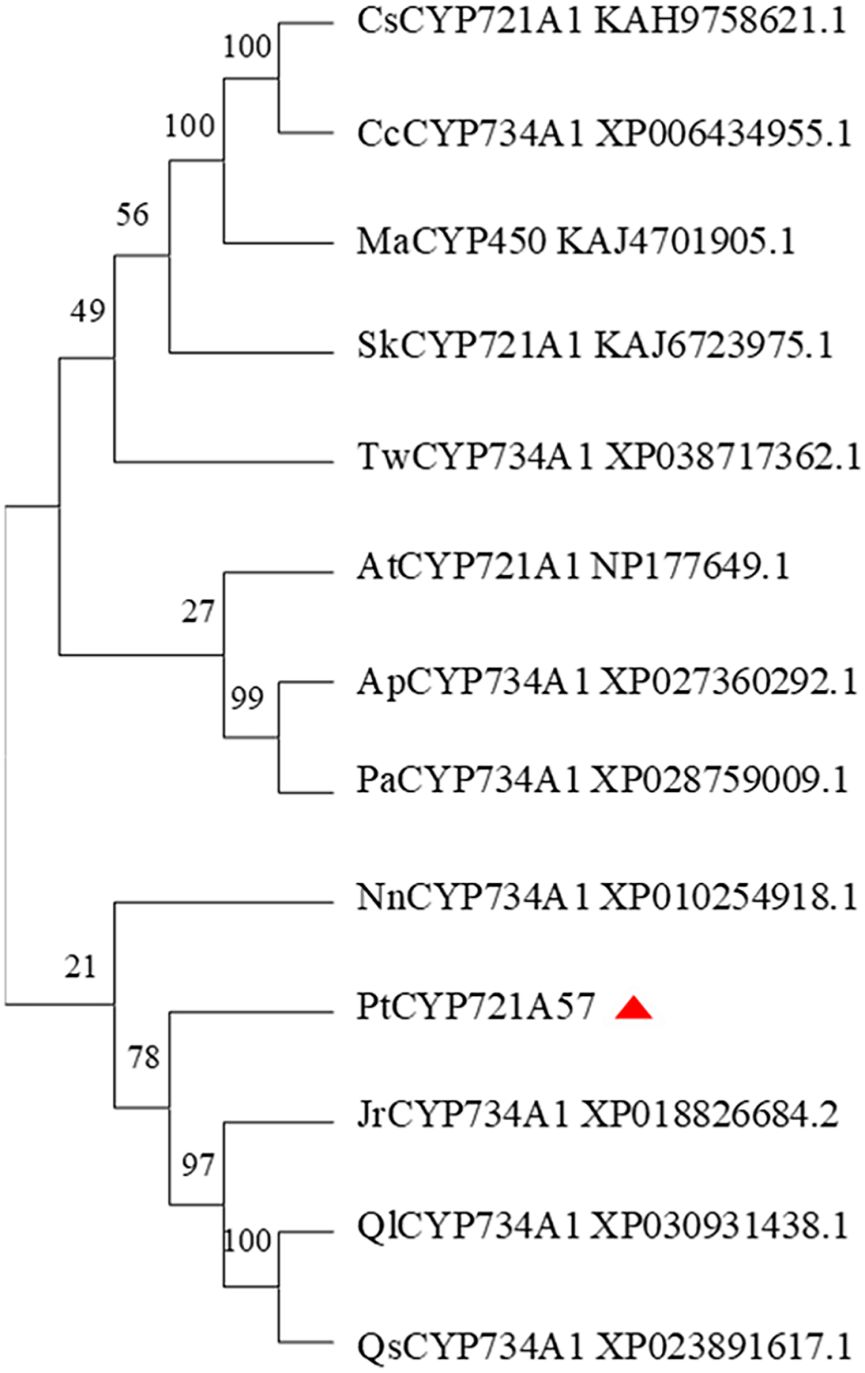
Expression pattern of *PtCYP721A57* in response to hormones (A) and stress treatments (B). CHT, Chitosan; ABA, Abscisic acid; Mock groups in A and B are the corresponding hormone and stress treatment mock groups, respectively. Different lowercase letters (a, b, c) above the columns represent statistically significant differences among different hormones and different stresses; different capital letters represent statistically significant differences between different processing times (*P* < 0.05).

Figure 9A illustrates the expression dynamics of the *PtCYP721A57* gene at different time points in *P. tenuifolia* seedlings under various stress and induction conditions, with 0 h (CK) representing the control. Under Mock treatment, *PtCYP721A57* expression initially decreased within 6 h, followed by an increase that peaked at 48 h (1.18-fold of CK). In the case of CHT induction, *PtCYP721A57* expression showed an initial decline, followed by an increase starting from 12 h, reaching its peak at 48 h (2.84-fold of CK), which was 2.50 and 1.63 times higher than that observed under Mock and CTS treatments at the same time point, respectively. Similarly, under ABA treatment, *PtCYP721A57* expression initially decreased and then increased, peaking at 48 h (1.75-fold of CK), which was 1.54 and 0.61 times higher than that observed under Mock and CHT treatments at the same time point, respectively.

Furthermore, Fig. 9B depicts the expression profile of *PtCYP721A57* under different stress treatments at various time points. Under PEG6000 treatment, the relative expression of *PtCYP721A57* gradually increased over the first 24 h, reaching its peak at 48 h (1.29-fold of CK), which was 1.05 and 1.89 times higher than that observed under Mock and NaCl treatments at the same time point, respectively. In contrast, NaCl treatment for 6 h significantly induced *PtCYP721A57* expression (3.16-fold of CK), which was 11.42 and 3.17 times higher than that observed under Mock and PEG treatments simultaneously.

## Discussion

The CYP450 family genes play crucial roles in the metabolic pathways of both endogenous and exogenous substances across a wide range of organisms. Currently, 127 CYP450 families have been found in plants, which are classified into 11 clusters showing distinct evolutionary branches on phylogenetic trees (Ghosh, 2017). These genes are known to catalyze various biological processes including growth, development, and the synthesis of terpenoids and sterols. In recent years, there has been increasing interest in the screening and exploration of *P450* family genes, particularly their functional roles in the biosynthesis pathways of triterpenoid saponins in medicinal plants (Das et al., 2023).

In this study, the complete cDNA sequence of the *PtCYP721A57* gene was cloned *via* PCR, followed by bioinformatics analyses. The results confirmed that *PtCYP721A57* possesses a full ORF and harbors a conserved domain characteristic of the P450 superfamily, thereby classifying it as a member of this family. The *PtCYP721A57* protein was identified as hydrophobic and stable. Subcellular localization assays indicated predominant localization of PtCYP721A57 within the endoplasmic reticulum. To facilitate further study, a prokaryotic expression vector, pET28a-PtCYP721A57, was constructed and introduced into BL21(DE3) cells for induction. The results demonstrated successful expression of the PtCYP721A57 recombinant plasmid at different temperatures and IPTG concentrations. Following sonication, gene expression was achieved in BL21(DE3), with significantly increased protein expression after IPTG induction. Phylogenetic tree analysis, mainly used to study the classification and evolution of gene family members, provided insights into the biological functions of closely related protein family members (Zhao et al., 2023). Molecular phylogenetic analysis of PtCYP721A57 and 10 other species within the CYP72 subfamily revealed its affiliation with the CYP72A subfamily. *PtCYP721A57* clustered primarily with GrCYP734A1, QlCYP734A1, and QsCYP734A1 from legumes, suggesting potential shared biological functions among these proteins. According to Biazzi et al. (2015), the synthesis of oleanane triterpenoid saponins typically involves CYP72 and/or CYP85 family clusters, implying the potential involvement of *PtCYP721A57* from *P. tenuifolia* in the biosynthesis of oleanane triterpenoid saponins.

P450 genes are pivotal in the regulation of various metabolic reactions in plants, and crucial for normal growth, development, and responses to environmental stresses. Regulation of P450 gene expression primarily occurs at the transcriptional level and exhibits tissue-specific specificity. Given the roots’ interaction with soil microecology, distinct expression responses of P450 genes are anticipated, potentially aiding plant adaptation and the synthesis of secondary metabolites (Gao et al., 2023). At present, the medicinal use of *P. tenuifolia* categorizes products into three specifications based on initial processing, with cortex-containing specimens commanding higher prices (Zhang et al., 2017; Wang, Peng & Hu, 2017). Analysis in this study detected higher tenuifolin levels in the cortex, correlating with prominent *PtCYP721A57* expression in this tissue. This suggests potential localization of tenuifolin within the phloem of *P. tenuifolia*. Endogenous hormones play important roles in signal transduction, regulating plant physiological and biochemical reactions, with *CYP450* gene expression closely related to these processes. *PtCYP721A57* responded significantly to ABA and CHT inductions, showing increased expression levels after hormone treatment. Additionally, *PtCYP721A57* responded to drought and NaCl stresses, indicating potential roles in mediating physiological and metabolic processes in *P. tenuifolia*. Through gene cloning and bioinformatics analyses, preliminary insights indicate that the *CYP721A57* gene may participate in downstream reactions of polygala oleanane triterpenoid saponin synthesis. However, the specific regulatory pathways and mechanisms need to be further studied. These findings enhanced our understanding of the functions of CYP450 genes involved in the synthesis of oleanane triterpenoid saponins in *P. tenuifolia*, providing scientific foundations for their regulation and genetic improvement.

## Conclusion

In this study, the *PtCYP721A57* gene from *P. tenuifolia* was cloned *via* PCR, and comprehensive analyses including bioinformatics, the accumulation of tenuifolin content across different tissues, response to hormones and abiotic stress, subcellular localization, and prokaryotic protein expression were conducted. These findings contributed to a deeper understanding of the pivotal role of the *CYP450* gene in the defense responses of *P. tenuifolia*. They also provided evidence for elucidating the biosynthetic pathways and regulatory mechanisms of *P. tenuifolia* saponins. Importantly, this study laid the groundwork for enhancing the quality of medicinal materials through genetic engineering and offered a basis for further investigations into the functions of *CYP450* genes in *P. tenuifolia*.

## Supplemental Information

10.7717/peerj.18089/supp-1 Supplemental Information 1 Raw qRT-PCR data.

10.7717/peerj.18089/supp-2 Supplemental Information 2 Hydrophobicity prediction analysis of amino acid sequence of PtCYP721A57.

10.7717/peerj.18089/supp-3 Supplemental Information 3 Prediction of phosphorylation sites of PtCYP721A57.

## Additional Information and Declarations

### Data Availability

The following information was supplied regarding data availability: The qRT-PCR data is available in the Supplemental File.
