# Supplementary figures and images for: Sequence characteristics, expression and subcellular localization of PtCYP721A57 gene from cytochrome P450 family in Polygala tenuifolia willd
Source: PeerJ. 2024 Oct 18;12:e18089. doi: 10.7717/peerj.18089 (PMC11493065; doi:10.7717/peerj.18089)

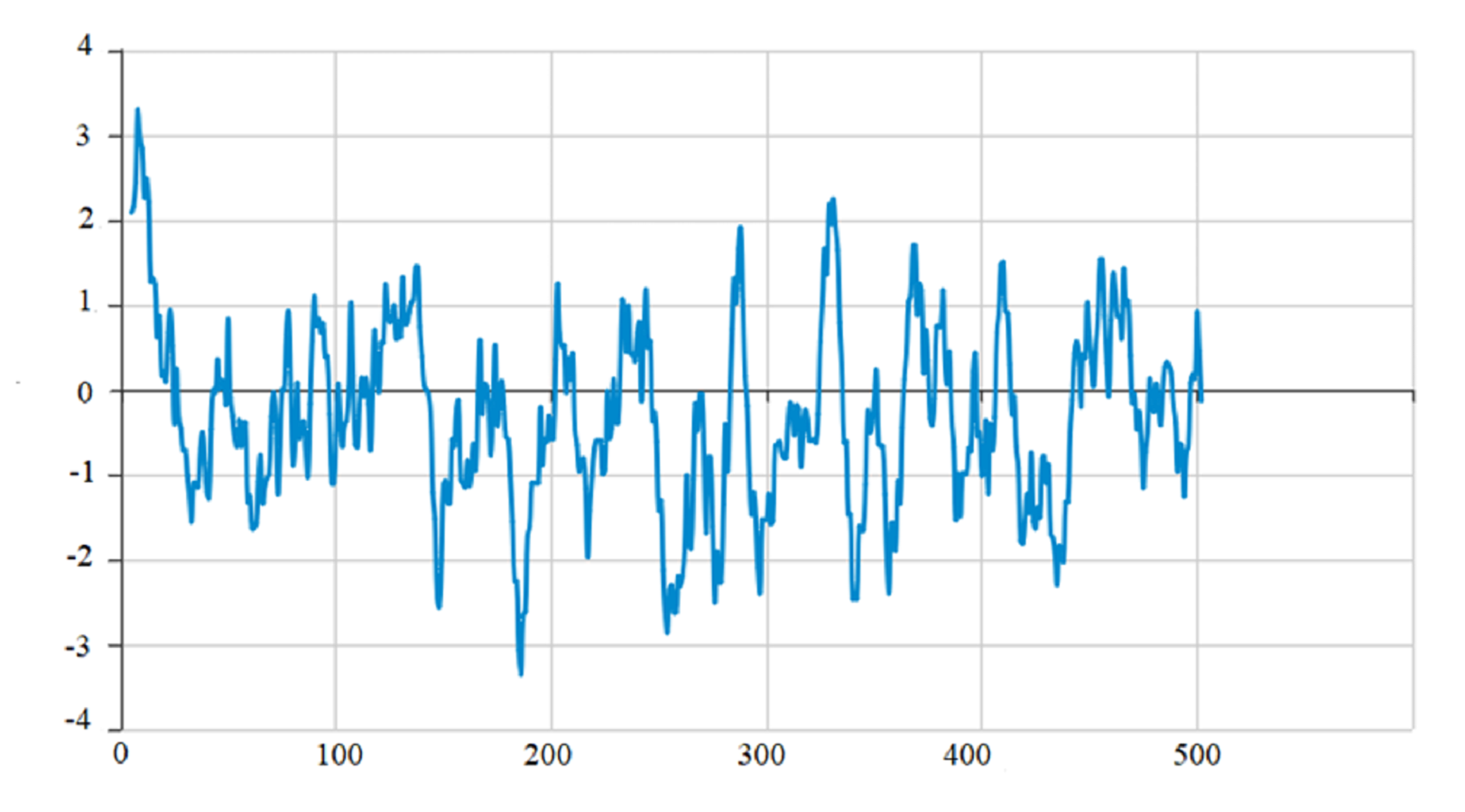

Supplement: Supplemental Information 2 [file peerj-12-18089-s002.png]

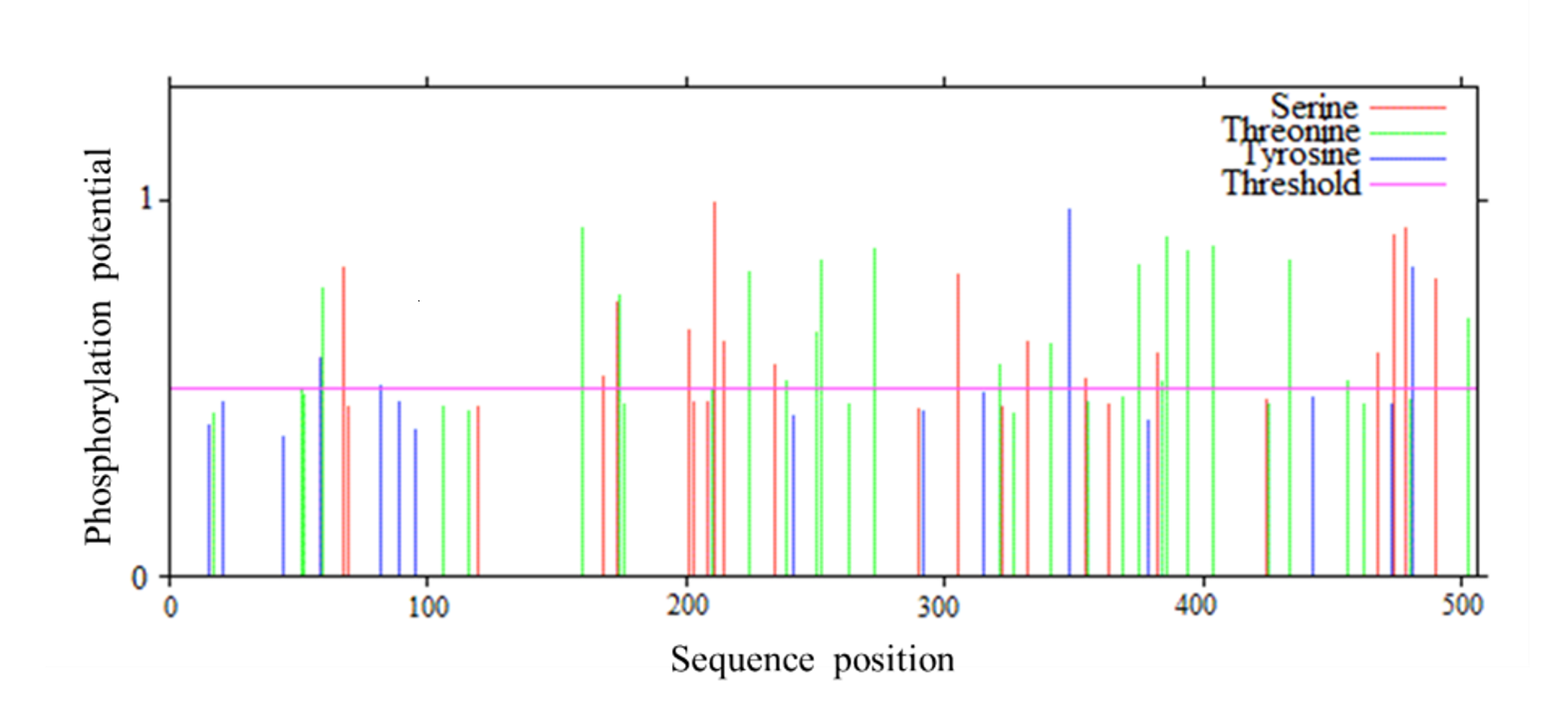

Supplement: Supplemental Information 3 [file peerj-12-18089-s003.png]
